# Rapunzel syndrome associated with trichotillomania and pica causing bowel obstruction in a 4 year-old-female: A rare case report from Nepal

**DOI:** 10.1016/j.ijscr.2024.110721

**Published:** 2024-12-09

**Authors:** Milan Pokhrel, Kundan Kumar Yadav, Rabi Khadka, Rupesh Verma, Dinesh Koirala, Geha Raj Dahal

**Affiliations:** a Maharajgunj Medical Campus, Institute of Medicine, Tribhuvan University, Kathmandu, Nepal; b Department of General surgery, Institute of Medicine, TUTH, Kathmandu, Nepal

**Keywords:** Rapunzel syndrome, Bezoars, Trichotillomania, Pica, Paediatric, Case report

## Abstract

**Introduction:**

Bezoars are an accumulation of human or vegetable fibers in the gastrointestinal tract which if extend to the small intestine are referred to as Rapunzel syndrome. They are primarily a psychiatric issue rather than a surgical condition and are associated with trichotillomania, with subsequent trichophagia, pica, and other psychiatric conditions usually seen in adolescent females. This case report describes an extremely rare diagnosis of Rapunzel syndrome in a 4-year-old female.

**Case presentation:**

A 4-year-old female presented with pain and a lump over the epigastric region for 1 week associated with an episode of vomiting, early satiety, and a history of ingestion of hair. There was a 4X4cm non-tender, firm, mobile, intraabdominal lump on examination. CT showed a moderately distended stomach with ill-defined heterogenous content extending up to the duodenum and suggesting bezoars. An open gastrotomy was performed to remove bezoars.

**Discussion:**

If bezoars extend beyond the pylorus to the small intestine, it is referred to as Rapunzel syndrome. Clinical features of RS include epigastric pain, epigastric lump, nausea and vomiting, early satiety, loss of appetite, halitosis, weight loss, and constipation. CT scan is the key to diagnosis and surgical intervention via open or laparoscopic approach is required. Psychiatric evaluation is essential for long-term management and prevents its recurrence.

**Conclusion:**

Bezoars are a rare cause of intraluminal intestinal obstruction and diagnosis should be considered if associated trichotillomania and pica are present in a paediatric patient who presents with abdominal pain, vomiting, early satiety, and weight loss.

## Highlights

• Rapunzel Syndrome is the extension of bezoars beyond the pylorus to the small intestine.
• Rapunzel syndrome usually occurs in adolescent females and is associated with trichotillomania with subsequent trichophagia, pica, and other psychiatric conditions.
• CT scan, Endoscopy, and USG help in diagnosis. Surgery for complete removal should be followed by long-term psychiatric follow-up to prevent recurrence.

## 1 Introduction

Bezoars are an accumulation of human or vegetable fibers in the gastrointestinal tract. The word “Bezoar” is derived from the Arabic word “bedzehr” or the Persian word “padzhar” which means “protecting against a poison” [1]. Bezoars can be of various types, the most common being phytobezoar (composed of plant fibers). Other types of Bezoars include trichobezoar which is composed of hair and Lacto bezoars (composed of milk formulas) [1,2]. They are usually confined to the stomach and if extension occurs beyond the pylorus to the small intestine, it is referred as Rapunzel syndrome [3]. It usually occurs in females and is associated with Trichotillomania, pica, learning disabilities, and other psychiatric conditions [2,3]. Trichotillomania means the compulsive desire of pulling one's hair [4] and Pica means compulsive eating of non-nutritive substances [5]. Here we report a rare case of a 4-year-old female with Rapunzel syndrome with pica and trichotillomania causing bowel obstruction.

This work has been reported in line with the SCARE Criteria [6].

## 2 Case presentation

**Fig. 1:**
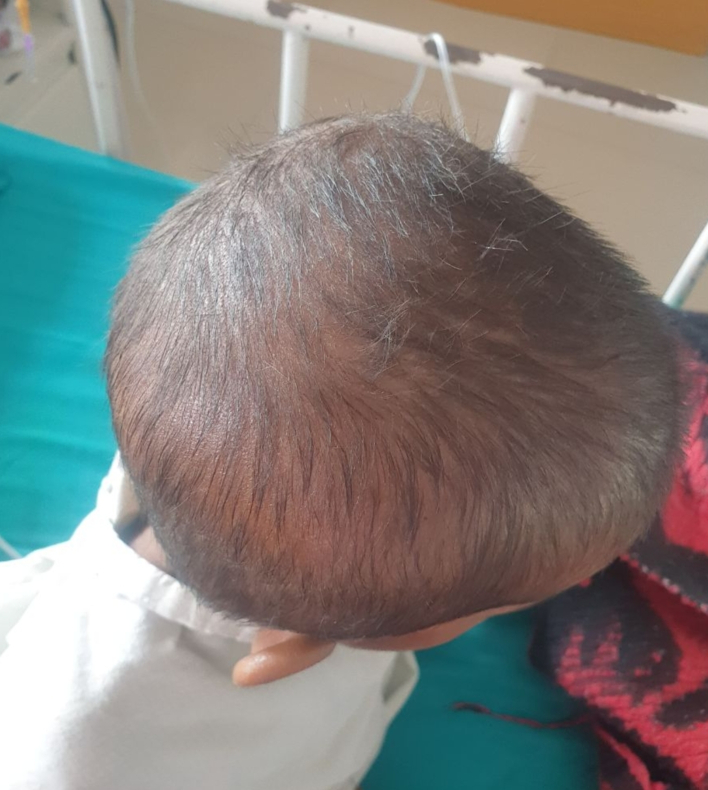
Generalized hair thinning due to Trichotillomania.

A 4-year-old female with a normal birth history, presented to the Emergency department of our center with complaints of abdominal pain (epigastric) for a week with episodes of vomiting and early satiety. Her parents noticed a palpable lump over the upper abdomen after the child complained of pain. There was a history of ingestion of hair 2 weeks before the current presentation. The parents complained that their child had similar hair-picking and hair-eating behavior resulting in a thinned-out hair (Fig. 1). There was no family history of eating disorders and the eating habits of the mother during pregnancy were normal.

**Fig. 2:**
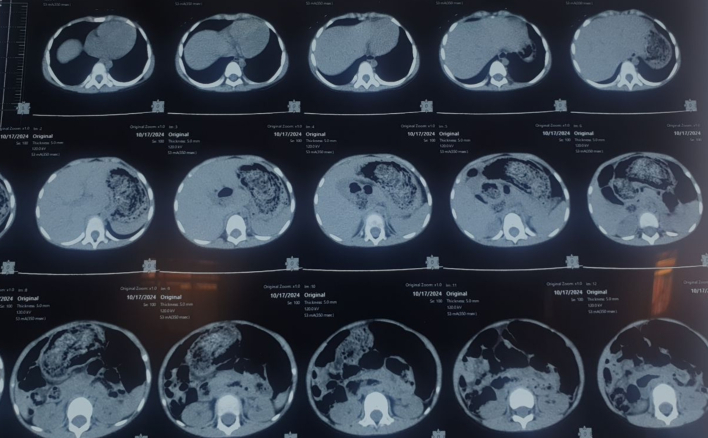
CT abdomen showing a moderately distended stomach with ill-defined heterogeneous content extending to the duodenum and suggesting Bezoars.

On general examination, pallor was present but features of dehydration were absent. The abdomen was soft, non-distended with a 4x4cm non-tender, firm, mobile intraabdominal lump in the epigastric region. With this history and examination, a diagnosis of gastric outlet obstruction secondary to trichobezoar was suspected. Complete Blood count showed a hemoglobin value of 10.7 mg/dl with low MCV, MCH, and MCHC suggesting microcytic hypochromic anemia. All other blood parameters were within normal limits except albumin (3.47 g/dl) and total protein (4.97 g/dl). CT scan of the abdomen shows a moderately distended stomach with ill-defined heterogeneous content within the lumen of the stomach and extending up to the duodenum suggesting bezoars (Fig. 2).

**Fig. 3:**
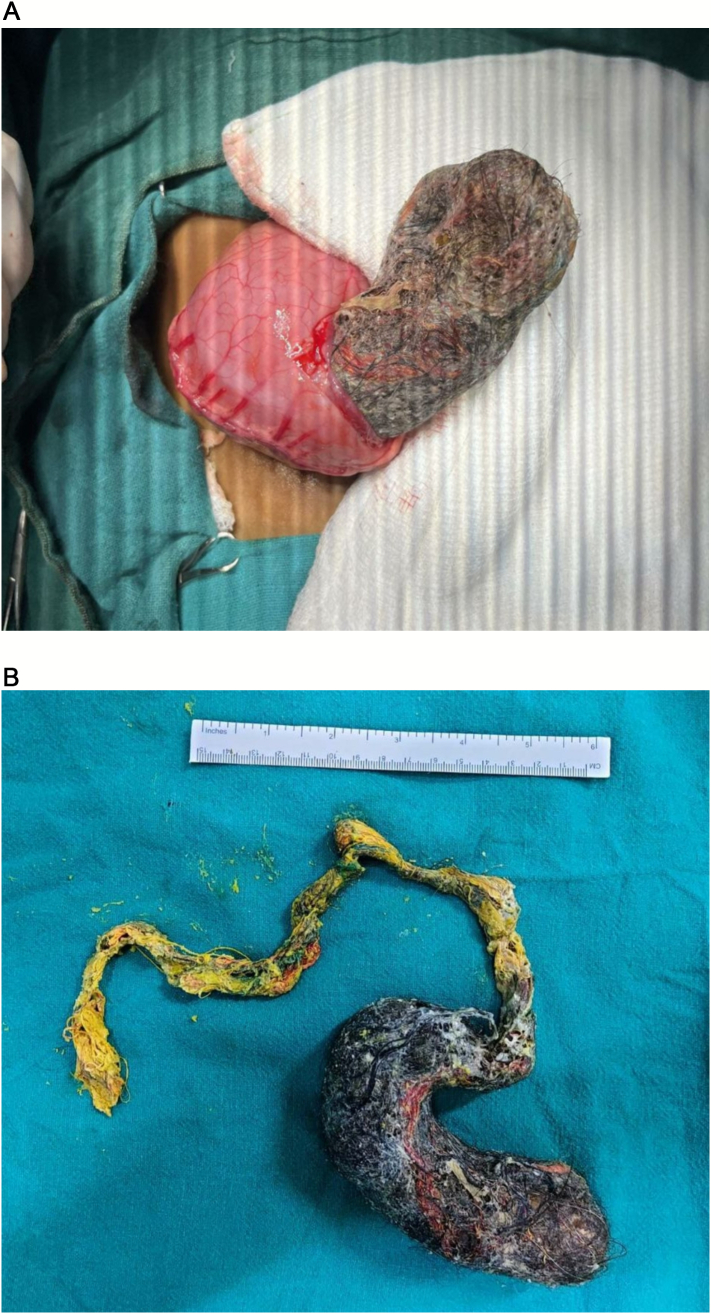
A. Intraoperative image of Gastrotomy during extraction of Bezoar. B. Stomach shaped mass of Bezoar with a tail extracted from the patient's stomach.

Gastrotomy (Fig. 3A) was done via the laparotomy approach, which revealed a stomach-shaped hair bunch mixed with thread with a tail extending beyond the duodenum (Fig. 3B). Her postoperative course was uneventful. During the hospital stay, the patient was evaluated for trichotillomania and trichophagia, but she did not come for regular psychiatry follow-up.

## 3 Discussion

Rapunzel syndrome derives its name from the Brothers Grimm's tale ‘Rapunzel’, written in 1812. The tale describes the story of a princess with long blonde hair trapped in a tower by an enchantress. It was first reported by Vaughan in 1968 [7]. It is common in females (90 % of cases), with the highest incidence between 10 and 19 years of age [7]. It is extremely rare in children with only 5 cases of 4 years or less among 78 reports of Paediatric Rapunzel syndrome [8]. Our case discusses a rare incidence of Rapunzel syndrome in a female child aged 4 years.

Clinical manifestations of RS are nonspecific and the patient may present with epigastric pain, epigastric lump, nausea and vomiting, early satiety, loss of appetite, halitosis, weight loss, and constipation [9]. Alopecia may be noted in patients but most of the patients deny the history of trichotillomania or trichophagia even when asked specifically [10]. Sometimes patient's condition may get complicated due to long-standing bezoar and gastric erosion, bleeding, acute pancreatitis, perforation, peritonitis, obstructive jaundice and malabsorption syndromes such as Iron deficiency anemia, megaloblastic anemia, protein loosing enteropathy may occur [9,11]. Lamerton's sign may be positive on examination, with a palpable, mobile, indentable lump in the upper abdomen [3]. The patient presented with features of bowel obstruction and associated features of trichotillomania and pica. Upon performing blood investigations, microcytic hypochromic anemia was found.

Diagnosis of RS is aided by Ultrasonography, Computed tomography (CT) scan, and upper gastrointestinal endoscopy [8]. In USG, bezoars are seen as an arcuate hyperechoic endoluminal formation with a posterior shadow cone [7]. Although UGI endoscopy is considered the gold standard for diagnosis, CT is more accurate in determining the presence of trichobezoars as well as the presence of small bowel obstruction. It also helps in therapeutic decision-making as well as choice of surgery. Mottled, non-homogenous space-occupying lesions in the lumen with low attenuation and air trapping is seen on CT scan [7,9,10]. Before presenting to our hospital, the patient had already undergone diagnostic evaluation in another center. USG was done, which revealed a normal study, so a CT scan was performed. This revealed ill-defined heterogeneous content within the lumen of the stomach and extending up to the duodenum, confirming the diagnosis of Rapunzel syndrome. Due to the patient's poor financial condition, investigations were not repeated, and a UGI endoscopy was also not performed.

Goals of management include complete removal of bezoars, treatment of complications if present and prevent its recurrence [7]. There are 2 major surgical approaches for the removal of bezoars: laparotomy and laparoscopy. Although laparoscopy is easier and less invasive, open laparotomy is the preferred surgical approach for large bezoars and Rapunzel syndrome [12]. Endoscopic removal of bezoars is nearly impossible most of the time due to their size. Even if bezoars are small in size, endoscopic removal is rarely done due to the risk of esophageal injury during the passage of the endoscope. Endoscopic removal is considered if surgery is contraindicated [7].

Long-term management of bezoars includes psychiatric evaluation of the patient and commencing behavior therapy to control trichotillomania [13]. Recurrence may occur due to a lack of effective psychiatric consultation requiring re-operation, thus special focus needs to be given for regular follow-up [13]. Unfortunately, our patient did not come for a follow-up psychiatric evaluation after getting discharged from the hospital; therefore, recurrence may occur in our case.

## 4 Conclusion

Rapunzel syndrome commonly affects adolescent females with some underlying psychiatric disorder. If a patient with features of intestinal obstruction also has trichotillomania, then the diagnosis of Rapunzel syndrome should be considered. Early diagnosis via thorough clinical history and examination and supported by investigations such as CT scan, endoscopy, or both helps prevent complications. Surgical management should be followed by long-term psychiatric follow-up to prevent recurrence.

## Consent

Written informed consent was obtained from the patient's parents for publication of this case report.

## Ethical approval

Ethical approval is not required for case reports in our institution.

## Guarantor

Milan Pokhrel

## Research registration number

Not applicable

## Funding

No funding for this research.

## Author contribution

Milan Pokhrel: Conceptualization, Writing - original draft, Writing - review & editing

Kundan Kumar Yadav: Writing - original draft, Writing - review & editing

Rabi Khadka: Conceptualization, Writing - original draft, Writing - review & editing

Rupesh Verma: Writing - review & editing

Dinesh Koirala: Writing - review & editing

Geha Raj Dahal: Writing - review & editing

## Conflict of interest statement

No conflicts of interest.
